# Nuclear Receptors in Myocardial and Cerebral Ischemia—Mechanisms of Action and Therapeutic Strategies

**DOI:** 10.3390/ijms222212326

**Published:** 2021-11-15

**Authors:** Joanna Rzemieniec, Laura Castiglioni, Paolo Gelosa, Majeda Muluhie, Benedetta Mercuriali, Luigi Sironi

**Affiliations:** 1 Department of Pharmaceutical Sciences, University of Milan, 20133 Milan, Italy; laura.castiglioni@unimi.it (L.C.); Paolo.Gelosa@guest.unimi.it (P.G.); majeda.muluhie@unimi.it (M.M.); luigi.sironi@unimi.it (L.S.); 2 Laboratory of Neuropharmacology and Epigenetics, Department of Pharmacology, Maj Institute of Pharmacology Polish Academy of Sciences, 31343 Krakow, Poland; 3 Centro Cardiologico Monzino IRCCS, 20138 Milan, Italy; benedettamercuriali@gmail.com

**Keywords:** estrogen receptors, aryl hydrocarbon receptor, peroxisome proliferator-activated receptors, brain, heart, myocardial infarction, stroke, selective estrogen receptor modulators, selective aryl hydrocarbon receptor modulators

## Abstract

Nearly 18 million people died from cardiovascular diseases in 2019, of these 85% were due to heart attack and stroke. The available therapies although efficacious, have narrow therapeutic window and long list of contraindications. Therefore, there is still an urgent need to find novel molecular targets that could protect the brain and heart against ischemia without evoking major side effects. Nuclear receptors are one of the promising targets for anti-ischemic drugs. Modulation of estrogen receptors (ERs) and peroxisome proliferator-activated receptors (PPARs) by their ligands is known to exert neuro-, and cardioprotective effects through anti-apoptotic, anti-inflammatory or anti-oxidant action. Recently, it has been shown that the expression of aryl hydrocarbon receptor (AhR) is strongly increased after brain or heart ischemia and evokes an activation of apoptosis or inflammation in injury site. We hypothesize that activation of ERs and PPARs and inhibition of AhR signaling pathways could be a promising strategy to protect the heart and the brain against ischemia. In this Review, we will discuss currently available knowledge on the mechanisms of action of ERs, PPARs and AhR in experimental models of stroke and myocardial infarction and future perspectives to use them as novel targets in cardiovascular diseases.

## 1. Introduction

Cardiovascular diseases (CVDs), with nearly 18 million estimated deaths globally represent the first cause of death worldwide, whereas stroke ranks second cause of death on this infamous list [1].

Heart and brain are organs with low ability for regeneration and damage of these tissues is irreversible. Myocardial infarction induces apoptosis, necrosis, autophagy and oxidative stress of cardiomyocytes [2]. Similarly, during a stroke, brain cells die mainly because of excitotoxicity, necrosis, apoptosis, autophagy and reactive oxygen species (ROS) overproduction [3]. In clinical practice, the main treatments for myocardial infarction are anti-platelet and thrombolytic therapies, angioplasty, stenting and coronary artery bypass surgery [4]. Instead, the approved treatment for stroke is the reperfusion therapy such as the recombinant tissue plasminogen activator (rt-PA) or endovascular mechanical thrombectomy (MT). In Japan, edaravone, an antioxidative radical scavenger is also used for the treatment of acute ischemic stroke [5]. However, it is needed to underline that the above mentioned treatments must be administered up to 6 h after symptoms onset for thrombolytic agents, up to 6–8 h for endovascular MT and up to 6–72 h for edaravone [5,6]. Since already existing treatments against myocardial infarction and stroke have a very narrow therapeutic window and a long list of contraindications, there is still an urgent need to find a more effective and safer therapeutic strategies to protect myocardial and brain cells against hypoxia/ischemia. Relevantly, epidemiological studies demonstrated that women are better protected against myocardial infarction and stroke than age-matched men [7,8]. However, the risk of myocardial infarction or stroke increases after menopause period [7,9]. The reason could be ascribed to the decreasing level of circulating estrogens, observed during aging, which are not only involved in the regulation of reproductive system, but also in the control of growth, differentiation and function of many tissues including heart and brain [10,11].

Estrogens exert genomic and non-genomic actions binding to one of the three subtypes of estrogen receptors (ERs), estrogen receptor alpha (ERα), estrogen receptor beta (ERβ) and G-protein coupled estrogen receptor-1 (GPER-1). Several in vitro and in vivo studies showed that estrogens acting via ERs exert cardioprotective and neuroprotective effects through anti-apoptotic, anti-inflammatory and anti-oxidant mechanisms [12,13,14,15]. It has been also shown that estrogen receptors interact with peroxisome proliferator-activated receptors (PPARs) [16] and with aryl hydrocarbon receptors (AhR) [17,18]. For example, ERα binds to PPAR response element and represses its transactivation [16]. AhR has been reported to inhibit ERs activity through the binding to the inhibitory xenobiotic response element (iXRE) presented in ERs target genes, squelching of shared coactivators or increased proteasomal degradation of ERs [18]. Therefore, in this Review, we will particularly focus on the role of ERs, AhR and PPARs in cardio-, and neuroprotection during hypoxia/ischemia in preclinical studies.

## 2. Targeting Estrogen Receptors as Potential Therapeutic Strategy in Myocardial Infarction and Stroke

**Figure 1:**
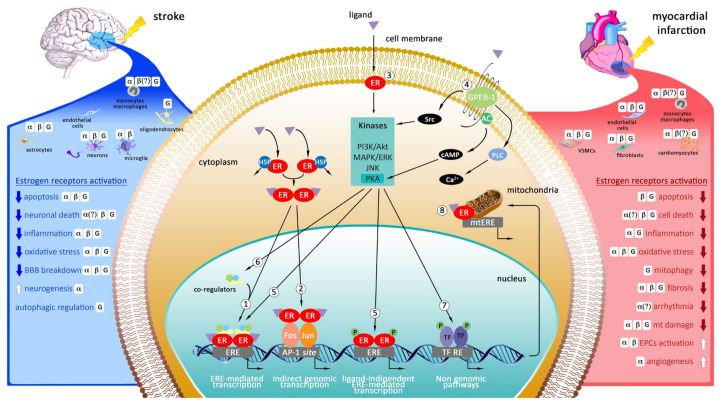
Schematic model representing the cellular localization, the molecular mechanisms and the effects of estrogen receptors activation after cerebral and cardiac ischemia. Genomic pathway: estrogen agonist directly induces dimerization of ERs and translocation to the nucleus. The complex ligands-ERs can (1) bind the estrogen-response-element (ERE) of gene promoters and induce target gene transcription, or (2) interacts with other transcription factors (TF) (e.g., Fos and Jun). Non-genomic pathway: estrogen agonist binds cell membrane ERs (3) or GPER-1 (4) triggering rapid cytosolic phosphorylation cascades through membrane-associated proteins. These kinases can phosphorylate and activate either ERs (5), both in a ligand-dependent and in a ligand-independent manner, or its associated co-regulators (6), enhancing or inhibiting the genomic action of ERs. Activated kinases can also affect gene transcription through phosphorylation of several TFs (7). In addition, estrogen-agonist activates ERs located in mitochondria, regulating mitochondrial function (8).

The two classic nuclear estrogen receptors, ERα and ERβ, are encoded by the *ESR1* and *ESR2* genes, which are located on chromosome 6 (6q25.1) and 14 (14q23.2), respectively [19,20]. The ERβ shows a high homology to ERα in the DNA-binding domain (more than 95% amino acid identity) and in the C-terminal ligand-binding domain (~55% amino acid identity) [21]. However, due to the splicing mechanism, the two subtypes of receptors may have different isoforms. In human, ERα has at least three isoforms of particular significance, while ERβ has at least five different isoforms [22]. These receptors bind 17β-estradiol (E2) and the other physiological ligands with similar affinity in their ligand-binding domains, except for 17α-estradiol [23]. The cellular relative concentrations of ERα and ERβ significantly influence cell’s response to various ligands. Estrogen can also activate GPER-1, a plasma membrane receptor encoded by the GPER1 gene, which is located on chromosome 7 (7p22.3) [24]. ERα and ERβ are mainly localized in cytosol and nucleus but were also found at the cell membrane, whereas GPER-1 is localized only within cell membrane. ERα, ERβ and GPER-1 are widely expressed in different tissue types and regulate important physiological functions, including reproductive, cardiovascular, muscular, and the central nervous system (CNS) [24,25]. The cellular localization, the mechanisms of action and the protective effects of estrogen receptors during heart and brain ischemia are described on the Figure 1.

### 2.1. Cellular Localization of Estrogen Receptors in the Heart

Cells expressing ERα and ERβ are present in the neonatal and adult heart [26,27], both in the ventricles and atria [28]. Cardiomyocytes, cardiac fibroblasts [29], endothelial cells [30], vascular smooth muscle cells (VSMCs) [31] and monocytes [32] were shown to express both ERα and ERβ, but recent papers doubt the expression of ERβ in cardiomyocytes and in monocytes [33,34,35,36]. The expression and localization of ERs are modulated in a disease-dependent manner. Indeed, in patients with aortic stenosis or heart failure, the myocardial expression levels of ERα and ERβ were increased [27,37], whereas no change was observed after myocardial infarction (MI) in mice [38]. GPER-1 is strongly expressed in all the chambers of the human heart and also in the atrioventricular sinus, avriet dextra, and aorta, but is not present in the atrioventricular node and apex [39]. GPER-1 is present in cardiac myocytes [40], fibroblasts [41], mast cells [42], VSMCs [43], endothelial cells [44] and monocytes [36].

### 2.2. Cellular Localization of Estrogen Receptors in the Brain

Cells expressing ERα, ERβ, and GPER-1 are localized throughout the brain, from the most rostral regions of the forebrain to the cerebellum. ERα and ERβ are present in neurons, both at axon terminals, in association with synaptic vesicles, and in dendritic spines [45], in astrocytes, near the spines of pyramidal cells, and in microglia [45,46]. ERβ is more abundant in the cortex and hippocampus than ERα, where it is present in pyramidal neurons and in interneurons. ERs were also found in cerebellum and hypothalamus [47]. GPER-1 is mainly expressed in the cerebral cortex, hippocampus, hypothalamus, striatum, and substantia nigra [47], and is localized in neurons, astrocytes and oligodendrocytes [48]. Interestingly, the expression of ERs and GPER-1 were reported to change across the estrous cycle and to show sex differences [45,48].

### 2.3. Genomic and Non-Genomic Mechanisms of Action of Estrogen Receptors

Estrogen-dependent signaling can be classically divided into genomic and non-genomic, although it is now widely established that there is a convergence of these pathways. Genomic action of ERs includes binding of ligand to the receptor, dimerization (ERα-ERα, ERβ-ERβ or ERα-ERβ dimers), translocation of dimers from cytoplasm to the nucleus, binding to the estrogen response elements (ERE) in the promoters of target genes and regulating gene expression [49]. Several studies have shown that ERs can also influence gene expression without binding directly to ERE. Indeed, ERs can interact with activator protein 1 (AP-1) transcription factor complex such as Fos and Jun proteins [50]. ERs can also exert non-genomic actions that are too rapid to be accounted for the regulation of gene expression and protein synthesis. Non-genomic actions are mediated by membrane-associated ERs and are related to the activation of pro-survival kinases such as PI3K/Akt and MAPK/ERK, attenuation of the pro-apoptotic pathway JNK [51], and mobilization of intracellular calcium [52]. Relevantly, the activation of these signaling transduction pathways by ERs can influence the genomic action of ERs themselves. Indeed, several kinases regulate the activation of ERs in both ligand-dependent and ligand-independent manner [53]. Among these, MAPK can phosphorylate and activate either ERβ or its associated coregulators, enhancing the genomic action of ERβ [52,53]. Furthermore, depending on which amino acid residues of ERα are phosphorylated, ERα-DNA binding could be increased or inhibited, leading to altered gene transcription [53]. Taking into account the above study, the convergence of non-genomic and genomic actions at multiple levels ensure an extremely high degree of control of gene transcription by ERs.

Localization of ERα and ERβ within mitochondria and in the mitochondrial membrane provides additional actions of estrogens [53]. To date, the mechanisms by which estrogens regulate mitochondrial function are not clearly understood. It has been shown that estrogens regulate transcription of nuclear respiratory factor-1 (NRF-1), peroxisome proliferator-activated receptor-gamma coactivator 1 (PCG-1), or mitochondrial transcription factor A (TFAM) which are critical for mitochondrial biogenesis and mitochondrial electron transport chain complexes [54]. It was also demonstrated that ERs can directly interact with mitochondrial ERE (mtERE) and in turn regulate mtDNA transcription [55].

The membrane GPER-1 receptor, formerly known as the G protein-coupled orphan receptor GPR30, has been shown to induce rapid signaling cascades following estrogens binding. Once activated, GPER-1 initiates several effectors, including c-Src and adenylate cyclase, which leads to increase of cAMP level and to the activation of prosurvival MAPK, PI-3K/Akt, and CREB pathways [56]. This mechanism is observed in neurons and in cardiomyocytes [57,58,59]. Interestingly, in astrocytes the activation of GPER-1 is associated with cell death via the activation of Phospholipase C (PLC) pathway and rise in intracellular calcium levels [60].

In addition, estrogen signaling is also tightly connected to epigenetic mechanisms. Several studies showed that estrogens could either induce demethylation of DNA resulting in epigenetic upregulation of downstream targets or methylation of DNA with subsequent downregulation of target genes [52]. Interestingly, the methylation level of *Esr1* decreased in female but not in male rats following middle cerebral artery occlusion (MCAO) [61]. This results were confirmed in women undergoing large-artery and cardio-embolic stroke who showed lower *ESR1* methylation levels in peripheral blood compared to the controls [62].

### 2.4. The Role of Estrogen Receptors in Myocardial Infarction

#### 2.4.1. ERs Modulation in Experimental Models of Myocardial Infarction

To assess the specific role of ERs in the pathophysiology of MI, several studies using ERs knock-out (KO) mouse or transgenic mouse models with ERs-overexpression have been carried out. Study performed on male and female ERα-KO mice, subjected to global myocardial ischemia/reperfusion (I/R), showed controversial results. Male ERα-KO mice subjected to global myocardial I/R, developed more severe cardiac damage, had a higher incidence of ventricular arrhythmias and showed a marked mitochondrial damage than wild-type (WT) mice, suggesting a cardioprotective role of ERα [63]. There results were not confirmed by another study, where no difference between ERα-KO male mice undergoing I/R and WT was observed. However, the female ERα-KO mice exhibited less functional recovery after I/R than WT female [64]. On the contrary, isolated Langendorff hearts from ERα-KO female mice exhibited I/R injury similar to that observed in WT females [65].

Studies in ERβ-KO mice suggest that this receptor plays a relevant role in the protection observed in the female heart. Indeed, isolated Langendorff hearts from ERβ-KO female mice exhibited significantly less functional recovery than WT female mice and were similar to WT male mice [65,66]. These ex-vivo results were corroborated by in vivo studies. At 8 weeks after permanent MI, ERβ-KO ovariectomized (OVX) female mice showed increased mortality and higher expression of pro-atrial natriuretic peptide (ANP) in left ventricle and in serum compared to WT mice [67]. In line, at two weeks after MI induced by left anterior descending (LAD) coronary artery ligation, estrogen treatment resulted in increased infarct size in ERβ-KO OVX mice respect to ERα-KO and WT OVX mice [68]. One possible mechanism of cardioprotection related to ERβ is the anti-apoptotic action. Indeed, ERβ-KO female mice undergoing MI had affected myocardial PI3K and Akt activation, which was associated with increased expression of caspase-3 and -8, as well as decreased Bcl-2 levels compared with wild-type (WT) mice. These effects were not observed in ERβ-KO male mice [66]. Luo and colleagues [69] observed that in ERα-KO, ERβ-KO and ERα/ERβ-double KO female mice the infarct volume was larger than in WT mice. Moreover, KO mice had reduced mitochondrial activity.

The role of ERs was also evaluated in inducible transgenic mice with cardiomyocyte-specific ERs-overexpression, making possible to discriminate the effect of cardiac ERα and ERβ from ERs-mediated systemic effects. In female mice ERα-overexpression improved functional myocardial adaptation, reduced collagen type-I and -III gene expression and collagen deposition, and induced phosphorylation of JNK signaling pathway 2 weeks after MI induced by permanent LAD ligation. Furthermore, ERα-overexpression enhanced angiogenesis, lymphangiogenesis and neovascularization in the peri-infarct area of female and male mice [70]. A recent study demonstrated that improved neovascularization after MI could be promoted by the activation of ERα in endothelial progenitor cells (EPCs) via enhancing their homing and angiogenic capacity [71]. Relevantly, transplantation of estrogen-stimulated EPCs preserved cardiac function after MI, but this effect disappeared in EPCs pre-conditioned with the ERα antagonist methyl-piperidino-pyrazole (MMP) [71] or derived from ERα-KO mice and, to a lower extent, from ERβ-KO mice [72].

It has been demonstrated that transgenic ERβ (Tg-ERβ) mice were more protected against MI than WT mice. At 1 week after MI induced by coronary artery ligation, Tg-ERβ mice had improved cardiac function with reduced echocardiographic end diastole and end systolic posterior wall thickness, increased ejection fraction and fractional shortening. Furthermore, cardiac collagen deposition and mRNA expression levels of collagen I, α-SMA, and TGF-β were significantly lower in Tg-ERβ than in WT mice [73]. Similarly, inducible ERβ-overexpression was associated with attenuated left ventricular (LV) dilatation, smaller increase in heart weight, improved systolic and diastolic function in MI mice of both sexes at 2 weeks from LAD ligation. These effects were associated with less reduction in diastolic Ca^2+^-reuptake into the sarcoplasmic reticulum [74]. Taken together, these results suggest a key role of ERs expressed in cardiomyocytes in the cardioprotective effects elicited by estrogen in both sexes.

Further insights on the specific ERs-mediated beneficial effects of estrogen have resulted from the development of selective ERα and ERβ agonists. Administration of the ERα-agonist propyl-pyrazole-triol (PPT) was cardioprotective in several models of MI. In intact or OVX rabbits subjected to cardiac I/R, treatment with 17β-estradiol (E2) or PPT decreased infarct size, release of cardiac-specific troponin-I and plasma level of C-reactive protein at the end of the 4 h reperfusion period [75]. Additionally, in isolated Langendorff hearts from adult or aged rats, PPT, improved ischemic tolerance via nongenomic ER signaling. PPT preserved PKCε levels at the nucleus and mitochondria, and enhanced the expression of PKCε anchoring protein RACK2 [76]. In sedentary OVX female rats, treatment of 2 weeks with PPT inhibited the acute I/R-induced increase of creatine kinase-(muscle–brain) (CK-MB), plasminogen activator inhibitor-1 (PAI-1) and TNF-α plasma concentrations, but not of IL-6, IL-8 and cardiac-specific troponin I. Instead, PPT failed to improve inflammatory cell infiltration, disorganization of cardiac muscle fibers and the microscopic damage score [77]. In ex-vivo hearts isolated from female OVX rats and subjected to MI by coronary ligation, PPT induced Akt and eNOS phosphorylation. These effects were abolished by co-incubation with PI3K inhibitor (LY294002) [78]. This result suggested that the ERα-activated PI3K/Akt signaling is involved in the modulation of eNOS activity. In the study with the same experimental design, PPT, but not diaryl-propio-nitrile (DPN), resulted in attenuated cofilin phosphorylation, suggesting that ERα, but not ERβ, mediated the inhibitory effect of estrogen on cofilin phosphorylation and thus on cardiac fibrosis after MI [78]. Similarly to PPT, ERA-45, another ERα-selective agonist, administered for 5 days prior to I/R in OVX rats reduced infarct size, neutrophil infiltration and oxidative stress at the end of the 2 h reperfusion period [79].

The role of ERβ was largely investigated using the ERβ agonist DPN. In isolated and Langendorff perfused hearts of OVX mice subjected to normothermic global ischemia, pre-treatment with DPN for 2 weeks induced a better functional recovery, decreased infarct size, upregulated expression of protective genes (heat shock protein 70, the antiapoptotic genes, growth arrest and DNA damage 45β, and cyclooxygenase 2) and increased the S-nitrosylation (SNO) of cardiac proteins [80,81]. The lack of these effects in ERβ-KO mice or in mice pre-treated with L-NAME, a NOS inhibitor, suggests that estrogens protect the heart through activation of ERβ and NO/SNO signaling [81]. On the contrary, in isolated and Langendorff perfused hearts of adult, aged, or aged OVX female Fischer 344 subjected to I/R, acute treatment of DPN had no effect on functional recovery [33]. Recently, it has been shown that treatment with DPN, for 14 days before and 2 days after LAD ligation in MI male mice, reduced the infarct size and the serum levels of myocardial enzymes (CK, CK-MB and lactate dehydrogenase) leading to cardiac function improvement. In parallel, DPN protected cardiomyocytes from oxidative damage (reduced the protein levels of iNOS and MDA, and increased SOD and GPX) and apoptosis (increased Bcl-2 and reduced cleaved caspase-3), exerting its cardioprotective function through the Notch1/PI3K/Akt signaling pathways [82].

Although ERs are well known to be cardioprotective, their hormone-dependent action on peripheral tissue is a strong contraindication to introduce them as a treatment of MI. Therefore; selective estrogen receptor modulators (SERMs) could be a good alternative for estrogens to contrast this disease. SERMs are compounds that act as ERs agonists or antagonist in tissue-dependent manner [83]. For example, SERMs representatives tamoxifen, raloxifene and bazedoxifene may act as ERs agonists in the cardiovascular system [84,85,86], but they antagonize ERs in breast tissue [7,87]. Rayabarapu and Patel [88] showed that tamoxifen and raloxifene significantly reduced isoproterenol-induced infarction and hypertrophy in rats. Cardioprotective effect of raloxifene was also observed by Chung and colleagues [89] who showed that long-term treatment with the SERM protects OVX rats against MI-induced arrhythmias and cardiomyocytes apoptosis via suppression of nuclear factor-kappa B (NF-κB).

#### 2.4.2. GPER-1 Modulation in Experimental Models of Myocardial Infarction

GPER-1 was detected in human heart and its expression could be modulated under pathological conditions. In isolated and Langendorff perfused hearts of rats, hypoxia resulted in about 2.4-fold increase in *Gper1* mRNA compared to basal conditions [39,59]. Furthermore, within the first 30 min of reoxygenation there was a substantial increase of *Gper1* mRNA expression, reaching 10.3 fold under basal conditions [39]. The pre-treatment with G1, a selective agonist of GPER-1, significantly reduced infarct size and improved the functional recovery of the left ventricular developed pressure (LVEDP). These effects were lost when hearts were pre-treated with GPER-1 antibody [39]. Other studies showed similar results of G1 in Langendorff perfused hearts of male and female rats or male mice, and demonstrated that G1 exerts its protective effects through PI3K/Akt [58] and ERK pathway [90]. The role of ERK, but not of PI3K/Akt, in the GPER-1 mediated cardioprotection against hypoxia in Langendorff perfused hearts was confirmed using ERs-KO mice. The authors suggest that estrogens, binding to GPER-1, may initially trigger translocation of protein kinase C (PKC), which could directly or via activation of MEK_1/2_/ERK_1/2_ pathway increase phosphorylation of GSK-3β. Deactivation of GSK-3β results in the inhibition of mitochondrial permeability transition pore (mPTP) opening [91]. This last effect is highly relevant, since the opening of mPTP plays a crucial role in the mechanism of cell death after ischemia/reperfusion [92].

In addition to ex-vivo studies, the role of GPER-1 was also evaluated in in vivo studies. In OVX rats subjected to permanent MI, 4 weeks of treatment with G1 improved the long-term MI-induced remodeling, reducing cardiac hypertrophy and fibrosis through phosphorylation and activation of AKT and eNOS [78]. In OVX mice subjected to LAD ligation, G1 reduced myocardial infarcted area and cardiac fibrosis, inhibited apoptosis via stimulation of PI3K/Akt pathway and diminished inflammation via decreasing TNF-α and increasing IL-10 levels [93]. The cardiac induction of the anti-inflammatory cytokine IL-10 was also observed in OVX diabetic rats treated with E2 or tamoxifen [94]. In this study, however the effect was GPER-1 independent.

A recent work showed that acute estrogen treatment induces cardioprotective effects in male and OVX female rats subjected to cardiac I/R by GPER-1 activation. At 3 h from reperfusion, estrogen reduced the percentage of area at risk, increased mitochondrial membrane potential and Ca^2+^ retention capacity, and decreased the production of ROS. The estrogen-mediated cardioprotective effect was related to activation of the MEK/ERK, deactivation of GSK-3β and to the delay of mPTP opening. Moreover, estrogen reduced mitophagy via the PINK1/Parkin pathway involving LC3I, LC3II and p62 proteins. The role of GPER-1 was pointed to the lack of these effects in presence of G-15, a GPER-1-antagonist [95]. In isolated and perfused hearts subjected to I/R, G1 reduced infarct size and improved contractile recovery in both normotensive and hypertensive female rats at 2 h from reperfusion. Relevantly, these cardioprotective effects were abolished by specific inhibitors of PI3K/Akt-eNOS-MitoKATP channels and by DAPT. DAPT is an inhibitor of the γ-secretase, an enzyme required for the Notch1 cleavage and activation. The lack of protective effect of G1 in presence of DAPT was also observed in cardiac myoblasts H9c2 cells subjected to I/R. These results suggested that G1 counteracted cardiac damage through activation of PI3K/Akt/NOS/MitoKATP channel and Notch1 pathways [96].

### 2.5. The Role of Estrogen Receptors in Stroke

#### 2.5.1. ERs Modulation in Experimental Models of Stroke

It is well known that estrogens exert anti-apoptotic, anti-oxidative and anti-inflammatory actions in the CNS [14,97,98]. The direct effect of E2 on microglia is well documented in many experiments in vitro. For example, E2 were able to reduce the expression of the pro-inflammatory mediators *Il1b* and *Ccl5* and to increase the expression of the anti-inflammatory cytokine *Il10* in immortalized microglial BV-2 cells undergoing hypoxia [99]. Furthermore, the pre-treatment of LPS-stimulated microglial N9 cells with E2 increased the IL-10 and decreased the TNF-α and interferon-γ release from these cells [100]. In vivo experiments using ERs-KO mice have suggested that ERα and ERβ play distinct roles in neuroprotection. The first research ruled out a role of ERα in the estrogen’s neuroprotective activity. Indeed, neurological function and ischemic volume were similar in ERα-KO and WT mice subjected to transient cerebral ischemia [101]. However, this study had some limitations, since the mice utilized were gonad-intact and thus the estradiol concentrations in ERα-KO mice were dramatically higher than in WT mice. On the contrary, in OVX mice subjected to permanent cerebral ischemia and treated with E2, deletion of ERα resulted in abolishment of neuroprotective effects, whereas in ERβ-KO mice neuroprotection was maintained [102,103]. Furthermore, the expression of ERα and ERβ was differentially modulated by ischemia and E2 treatment [103,104], leading the authors to speculate that ERα may be fundamental in the protection against cell death, while ERβ may play a role in regeneration and neurogenesis. This hypothesis is not fully shared. Indeed, the silencing of ERβ via intracerebroventricular (i.c.v.) injection of ERβ-antisense inhibited the E2-mediated hippocampal protection in OVX rats subjected to transient cerebral ischemia [105]. Estrogens can also exert protective effect on very early stages of ischemic injury. A recent study showed that estrogen or DPN o PPT pretreatment protected brain endothelial (bEnd3) cells against oxygen and glucose deprivation/reoxygenation (OGD-R) injury. DPN and PPT increased OGD-downregulated levels of occludin and claudin-5. Silencing of ERα or ERβ with the use of specific siRNAs completely reversed the effects of DPN or PPT on the outcomes of OGD-R [106]. These data strongly suggest an involvement of estrogen receptors in maintaining BBB function during the stroke.

Apart from the study carried out in ERs-KO mice or cells, the neuroprotective potential of ERs is often confirmed by the use of specific ERs agonist. In OVX rats subjected to transient global cerebral ischemia, ERα selective agonists PPT elicited a pronounced protection of CA1 pyramidal neurons in approximately 40–50% of treated ischemic rats [104]. This result was in contrast with two other studies showing a lack of neuroprotective action of PPT in OVX mice with transient global ischemia induced by bilateral carotid artery occlusion [107] and in male mice with transient global ischemia induced by cardiac arrest [108]. This discrepancy could be explained by the different dose utilized or differences in performing ischemia. A recent study demonstrated that metastasis-associated protein 1 (MTA1), which is a chromatin modifier and transcriptional regulator, could be a factor linking ERα with apoptosis. The increase of MTA1 expression in mice after transient middle cerebral artery occlusion (tMCAO) promoted interactions between ERα and anti-apoptotic Bcl-2 which in turn diminished ischemia-induced brain damage [109]. In line, endogenous estrogen in proestrus protected female rats against I/R injury by an increase of ERα-dependent *Bcl-2* expression [110]. ERα could be also involved in the neuroprotection mediated by the inhibition of *miR-181a*. Indeed, *miR-181a* inhibition led to increase of *Esr1* expression that in turn resulted in decrease in infarct volume and improved neurological deficit score in OVX mice subjected to tMCAO. Furthermore, it decreased death in female astrocytes cell culture subjected to glucose deprivation [111].

Not only ERα agonists but also ERβ agonists may protect the brain against ischemia. In OVX mice with transient global ischemia induced by bilateral carotid artery occlusion, ERβ agonist DPN significantly reduced ischemic damage in the caudate nucleus and in the CA1 region compared with vehicle controls [107]. Similarly, in male mice with transient global ischemia induced by cardiac arrest, DPN reduced neuronal injury in the striatum and in CA1 field [108]. The periodic DPN treatment (every 48 h) improved post-ischemic learning and memory in OVX rats subjected to transient cerebral ischemia [105]. More recent studies showed that DPN diminished I/R evoked injury in OVX mice via inhibition of microglia, astrocytes and NF-κB-mediated neuroinflammation [112,113]. Moreover, specific ERβ agonist AC-131 helped to recover the neurological function in male rats with permanent focal ischemia induced by photothrombosis [114]. In OVX rats subjected to transient cerebral ischemia, specific ERβ agonist WAY 200070-3 elicited pronounced protection of CA1 pyramidal neurons in approximately 40–50% of treated ischemic rats [104]. Interesting results were also obtained in OVX mice with transient focal brain ischemia where DPN reduced the extravasation of endogenous immunoglobulin G (IgG), vasogenic edema, and the infarct volume [115].

Despite the well-documented, beneficial action of estrogens in experimental models of stroke their clinical use is limited because of the hormone-dependent action on peripheral tissue. Therefore, a good alternative for the use of estrogens in stroke treatment could be the use of selective estrogen receptor modulators. There are already some data showing that SERMs mimic the action of estradiol after experimental ischemia avoiding hormone-dependent risks. In rats subjected to transient or permanent MCAO (pMCAO) tamoxifen significantly reduced the infarct size and protected neurons against ischemia [116,117]. In OVX rats with pMCAO, tamoxifen reduced MCAO-elevated superoxide anion production, oxidative damage and caspase-3 activation, through increasing the levels of manganese SOD (MnSOD) and attenuating the elevation of pERK1/2 [118]. However, the involvement of ERs in the mechanism of action of tamoxifen is still unclear. In rats with tMCAO, the neuroprotection by tamoxifen was maintained when co-administered with the estrogen receptor antagonist ICI 182,780, suggesting a key role of its antioxidant activity but not of estrogen receptors [119]. In contrast, tamoxifen enhanced neuronal survival in OVX rats with tMCAO by modulating ERα-36, a variant of ERα, and activating the MAPK/ERK signaling pathway [120]. Similarly to tamoxifen also raloxifene and bazedoxifene protected mouse hippocampal and neocortical cells against hypoxia via ERα, but not ERβ and GPER-1 [121,122]. Raloxifene and tamoxifen preserved spine density in the cortex of OVX rats, but only raloxifene enhanced neurogenesis after tMCAO. The Authors suggested that similarly to estrogens, the SERMs may enhance excitatory synapse formation in cortical neurons via a non-genomic ER-mediated mechanism involving up-regulation of AMPA receptor by Akt and ERK signaling pathways [123]. Another SERM representative bazedoxifene mimicked action of estradiol in male rats with tMCAO [124] and improved neurological function via ERα and ERβ, but not of GPER-1 [125]. Similar effect was observed in diabetic rats after tMCAO [126], suggesting the involvement of ERs in the neuroprotective effects of bazedoxifene.

#### 2.5.2. GPER-1 Modulation in Experimental Models of Stroke

GPER-1 plays a relevant role in the acute neuroprotective effects of estrogen against ischemic injury. It was shown that the magnitude of neuroprotection observed in G1 treated OVX mice with tMCAO was indistinguishable from estrogen treated mice [127]. GPER-1 is involved in inhibition of neuronal deficit and inflammation after ischemic injury in OVX mice with tMCAO. Activation of this receptor reduced the infarct volume, improved the neurological deficit and alleviated neuronal injuries via inhibition of microglia activation and cytokine’s release [128,129]. In transient global ischemia in OVX rats, G1 inhibited inflammation decreasing the expression of NLRP3-ASC-caspase 1 inflammasome and IL-1β as well as NF-κB signaling. Intriguingly, G1 caused a robust elevation of endogenous anti-inflammatory factor IL1RA in neurons, likely enhancing CREB phosphorylation. Relevantly, IL1RA antisense oligodeoxynucleotide abolished the anti-inflammatory, neuroprotective, and anti-apoptotic effects of G1 [130]. The anti-inflammatory activity of GPER-1 was also corroborated by the fact that GPER-1 antagonist G-15 reversed the effect of estrogen and abolished the reduced serum level of IL-1β and TNF-α in rats with global cerebral ischemia [128].

In additions to anti-inflammatory effects, GPER-1 could mediate neuroprotection through different mechanisms. Indeed, G1 reduced the infarct volume and neurological deficit score in OVX mice with tMCAO, but these effects were abolished by simultaneous administration of PD98059, an ERK1/2 inhibitor, suggesting that ERK activation by GPER-1 is required to protect the neurons [131]. The involvement of GPER-1 in the neuroprotective effect of E2 was not confirmed in organotypic hippocampal slice cultures exposed to ischemia. Indeed, GPER-1 activation only facilitated the acute E2 mediated neuroprotection after OGD, since GPER-1 antagonist G-15 effected a slight but not significant reduction in neuroprotection elicited by E2 [132]. However, G1 could play an important role in protecting the cerebrovasculature against ischemia. In fact, in ex-vivo study, G1 significantly improved the ATP-vasodilation in both male and female rat vessels with hypoxia/reoxygenation (H/R) injury similar to pre-H/R dilations [133]. GPER-1 could also counteract BBB disruption after brain ischemia. In OVX rats with induced transient global cerebral ischemia, G1 decreased IgG extravasation and increased the levels of tight junctions (occludin and claudin-5) in the CA1 [134]. In male mice with transient global ischemia induced by cardiac arrest, G1 reduced neuronal injury in the hippocampal CA1 region and striatum via increased expression of the small conductance calcium-activated potassium channel 2 [135]. Finally, G1 reduced the neurological deficit scores and the infarct volume in WT mice with tMCAO, but these effects were completely blocked in conventional GPER-1 KO mice and only partially attenuated in astrocytic or neuronal GPER-1 KO mice. The authors suggest that astrocytic GPER-1 could play a key role in neuroprotection, via restoration of autophagic balance through the activation of p38 MAPK signaling pathway [136].

One research study showed sex-dependent protection of GPER-1 agonist. In male rats with tMCAO, G1 worsened functional outcomes and increased infarct volume, which was associated with higher level of cleaved caspase-3 in peri-infarct neurons. On the contrary, G-15 improved functional outcomes and reduced infarct volume after stroke in males. In OVX female mice, G1 reduced neurological deficit, apoptosis, and infarct volume, but had no effect in intact females [137]. The authors hypothesized that after cerebral ischemia GPER-1 may become the predominant estrogen receptor expressed in brain of male mice, whereas the ERα predominates in female mice.

## 3. Targeting of Peroxisome Proliferator-Activated Receptors (PPARs) as Potential Therapeutic Strategy in Myocardial Infarction and Stroke

**Figure 2:**
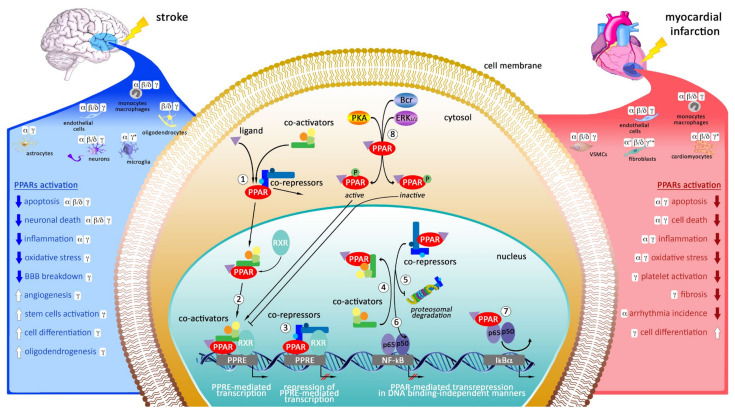
Schematic model representing the cellular localization, the molecular mechanisms and the effects of PPARs activation after cerebral and cardiac ischemia. Under unliganded state, monomer or dimer of PPAR is bound to multicomponent repressors (1). Ligand-dependent transactivation: ligand binding to either PPAR or RXR causes displacement of bound repressors, recruitment of co-activators and activation of gene transcription (2). Ligand-independent repression: PPARs bind to response elements in the absence of ligand and recruit multicomponent repressors that mediate active repression (3). Ligand-dependent repression is provided by several mechanisms: competition for a limiting pool of co-activators (4), inhibition of repressors clearance (5), direct interaction with other transcription factors (e.g., p65/p50) (6,7). Finally, several kinases can phosphorylate PPARs modulating its activity (e.g., PPAR-mediated transcription is increased by PKA, while is reduced by MAPK and Brc kinase) (8). ° the expression is increased after MI; * the expression is increased after LPS treatment.

PPARs are ligand-activated nuclear transcription factors, belonging to nuclear receptor superfamily and including PPAR-α, PPAR-β/δ and PPAR-γ subtypes [138]. They are encoded by *PPARA*, *PPARD*, *PPARG*, respectively. The *PPARA* gene is located on human chromosome 22q12.2-13.1 [139], and its expression is the highest in high-energy requiring tissues such as liver, heart, and skeletal muscle where it acts as a major regulator of the mitochondrial, peroxisomal, and microsomal fatty acid oxidation systems [140]. The *PPARD* gene is located on human chromosome 6p21.1-21.2 [139] and is highly expressed in adipose tissue, liver, heart, skeletal muscle, brain, kidney, colon, and vasculature [141]. Activation of PPAR-β/δ induces lipid catabolism and energy dissipation. Unlike PPAR-γ and PPAR-α, PPAR-β/δ is not easily targeted by currently available drugs because of its ubiquitous expression. Finally, the *PPARG* gene is located on human chromosome 3p25 [139] and is widely expressed in brown and white adipose tissues, and in the immune system, where it regulates glucose metabolism, cell differentiation, and immune and inflammatory responses [142]. The cellular localization, the mechanisms of action and the protective effects of PPARs during heart and brain ischemia are described on the Figure 2.

### 3.1. Cellular Localization of PPARs in the Heart

All three PPAR isoforms are found in the adult heart, with various levels of expression. PPAR-α and PPAR-δ are highly expressed at similar levels to those in other metabolically active tissues, such as the liver and skeletal muscle [143], while PPAR-γ is expressed at very low levels, approximately 2% of those in adipose tissue [144]. However, the cardiac expression of PPARs could change in pathological conditions. Indeed, PPAR-α expression and activity are diminished in cardiac tissue of pressure overload–induced hypertrophy murine model [145], in hypertrophic heart of spontaneously hypertensive stroke-prone rats [146] and in hypoxic cardiomyocytes [147] leading to a reduction in the capacity for fatty acid oxidation and increased rates of glucose utilization. Rats with diabetic cardiomyopathy presents decreased cardiac levels of PPAR-α and PPAR-β/δ [148,149], while those of PPAR-γ are increased [149]. Instead, the PPAR-α and PPAR-β/δ proteins level didn’t change after myocardial infarction in rats, while PPAR-γ showed a significant increase in the infarcted area [150].

Concerning the cellular localization of PPARs, several differences were observed among the three PPAR isotypes. In neonatal and adult rat cardiomyocytes, both PPAR-α and PPAR-β/δ were expressed comparably, while PPAR-γ is barely detectable [151]. However, PPAR-γ protein expression was strikingly increased in cardiomyocytes in the infarcted area of rat underwent LAD ligation [150]. In cardiac fibroblasts and myofibroblasts, PPAR-β/δ is higher expressed and more biologically active than PPAR-α and PPAR-γ [150,152]. Like the cardiomyocytes, the protein content of PPAR-γ was increased in connective tissue and in fibroblasts in the infarcted area of rats underwent LAD ligation [150]. Finally, all the three PPAR isotypes have been reported in the cells of vascular wall, such as smooth muscle cells, monocyte/macrophages and endothelial cells [153,154,155].

### 3.2. Cellular Localization of PPARs in the Brain

In adult murines, PPARs are ubiquitously expressed in all brain regions [156,157], although recent evidence has demonstrated brain region- and cell type-dependent differences in PPARs subtypes expression. As reported, mapping the PPARs isotype mRNA and protein in the adult mouse, the general order of abundance across all brain regions was PPAR-β/δ > PPAR-α ≥ PPAR-γ [157]. PPAR-α is strongly expressed in neurons, in the cell body and processes of astrocytes but weakly in microglia of the ventral tegmental area (VTA), prefrontal cortex (PFC), nucleus accumbens (NAC), or amygdala (AMY). Similarly to PPAR-α, PPAR-γ is more expressed in neurons than in astrocytes with the highest level in the NAC and the lowest in the PFC. Instead, PPAR-γ does not colocalize with microglia in the adult mouse brain. Finally, PPAR-β/δ is primarily localized in the nucleus of neurons, while it is not expressed by astrocytes in grey matter and in microglia [157]. In human brain, the pattern of PPARs expression is similar to the mouse brain [157]. In particular, PPAR-α colocalized with all cell types, while PPAR-β/δ and PPAR-γ colocalized with neurons and astrocytes, but not with microglia. Although the expression of PPAR-γ is not detectable in physiological condition in microglia, PPAR-γ may be tightly regulated and dependent on microglial functional state. Indeed, PPAR-γ expression was induced in microglia after LPS treatment or specific agonists [157]. On the contrary, PPAR-β/δ still was not expressed by microglia after lipopolysaccharides (LPS) treatment [157]. PPAR-γ and PPAR-β/δ are also expressed in oligodendrocytes, promoting survival and differentiation of precursor cells [158], while PPAR-α, PPAR-γ and PPAR-β/δ are also expressed in brain capillary endothelial cells, suggesting an involvement of the receptors in regulation of BBB [159,160,161].

### 3.3. Mechanisms of Action of Peroxisome Proliferator-Activated Receptors

Differently to the estrogen receptors, which create homodimers, PPARs form heterodimers with the retinoid X receptor (RXR) [162]. In the absence of ligand, PPARs/RXR heterodimer is bound to multicomponent repressors with histone deacetylase activity, such as the nuclear receptor corepressor (NCoR) and silencing mediator of retinoid and thyroid hormone receptor (SMRT), thereby inhibiting gene transcription [163]. A ligand binding triggers dissociation of corepressors from PPARs/RXR heterodimer, and recruitment of co-activators. The entire complex binds to peroxisome proliferator response elements (PPREs) located in the promoter region of target genes resulting in initiation of gene’s transcription [163].

However, it was also demonstrated that phosphorylation can modulate PPARs activity [164,165]. The protein kinase A (PKA)-induced phosphorylation of PPARs has a stimulatory effect on transcription in a ligand-independent and ligand-dependent manner [165], while MAPK and Brc kinases-induced phosphorylation deactivates PPAR-γ and reduce basal and ligand-dependent transcriptional activity [164,166]. PPARs can also inhibit gene expression in a DNA binding-independent manner, interfering with other transcription factors. First, PPARs could repress transcription in a ligand-dependent manner by competition for a limiting pool of co-activators with NF-kB and activator protein-1 (AP-1), leading attenuation of NF-kB and AP-1 target gene expression [167]. Second, PPARs could inhibit the expression of proinflammatory genes by direct interaction with NF-kB, Smad-3, AP-1, signal transducers and activators of transcription (STAT) proteins, preventing the binding to their response elements [167]. Third, PPARs can contribute to transrepression by either preventing the clearance of co-repressor complexes [168] or releasing co-repressors, which could allow co-repressor binding to NF-kB, eventually inhibiting NF-kB target gene expression [167].

### 3.4. The Modulation of PPARs in Experimental Models of Myocardial Infarction

PPARs are mainly involved in energy homeostasis and metabolic function, however there is increasing body of evidence on their cardioprotective potential [169,170]. Indeed, it has been shown that the PPAR-γ agonist-pioglitazone protected the mouse heart from myocardial injury by antagonizing monocyte/macrophage-mediated inflammation and promoting cardiac healing [171]. Moreover, theacrine (alkaloid derived from Chinese tea) inhibited myocardial infarction-evoked fibrosis via stimulation of *PPARG* and *SIRT3* expression [172]. Similarly, plant-derived chrysin attenuated the MI-induced fibrosis in rats via up-regulation of PPAR-γ, down-regulation of matrix metalloproteinases-2 and -9 and inhibition of the NF-κB pathway [173]. Another natural compounds such as qiliqiangxin, apigenin or curcumin also protected cardiomyocytes against myocardial infarction via activation of PPAR-γ [174,175,176]. Shen and colleagues [177] showed that knock-out of PPAR-γ in mice myeloid cells led to cardiac hypertrophy and increased myocardial infarct size. This was correlated with induction of oxidative stress and cardiac inflammation. Interestingly vitamin D exerted anti-inflammatory and anti-oxidant effects in rat model of myocardial infarction via PPAR-γ [178].

Growing body of evidence demonstrates importance of miRNAs in the regulation of gene expression. It has been shown that PPAR-γ is a target gene of *miR-130*. In cardiomyocytes cell line H9C2 undergoing hypoxia, downregulation of *miR-130* promoted PPAR-γ-mediated cardioprotection by inhibiting NF-κB-mediated inflammation and TGF-β1-mediated fibrosis. These results were also confirmed in vivo [179]. Zhu and colleagues [180] showed that *miR-292-5p* downregulation protects mice against myocardial ischemia through activation of the PPARα/PPAR-γ signaling pathway.

Platelet activation is one of the major pathophysiological mechanisms that underlie I/R injury. It has been demonstrated that humans and rodents undergoing acute myocardial infarction have lower level of PPAR-γ in platelets following by mitophagy activation and an increase in mitochondrial function. Improved mitochondrial function in turn lead to platelet aggregation and formation of microthrombus thus, the inhibition of this process may cause protective effects. Indeed, Zhou and colleagues [181] showed that melatonin inhibited platelet activation via increasing of the PPAR-γ level, blocking mitophagy, platelet hyperactivity, and cardiac I/R injury. Another therapeutic strategy in myocardial infarction is related to the replacement of dead cardiomyocytes or regeneration of the heart muscle. Although, there are studies showing positive effects of stem cells administration to the injured tissue [182,183], stem cells cannot ensure full regeneration of the heart muscle and in turn recovery of heart functions. Therefore, it is crucial to combine stem cells therapy with pharmacological treatments. Hou and colleagues [184] showed that rats simultaneously treated with mesenchymal stem cells (MSCs) and pioglitazone after MI significantly improved cardiac functions via stimulation of PPAR-γ-regulated *Cx43* expression and inhibition of TGF-β1/Smad signaling pathway. This kind of strategy seems to be promising considering also that PPAR-γ is crucial for cardiomyocyte differentiation [185].

Apart from PPAR-γ, also PPAR-α seems to be involved in cardioprotection against myocardial infarction. Indeed, it has been shown that natural compound called raspberry ketone suppressed isoproterenol-induced cardiac infarct size, oxidative stress and inflammation in rats via activation of PPARα [186]. Administration of the PPAR-α agonist WY-14643 inhibited myocardial infarction and reperfusion-induced arrhythmia in rat model. PPAR-α activation protected also H9C2 cells against hypoxia-reoxygenation via increased *Ucp3* expression and attenuation of ROS production [187]. However, there are also data showing that overexpression of PPAR-α in mice heart led to cardiomyocytes cell death during ischemia/reperfusion [188]. Similarly, conditional overexpression of PPAR-β/δ in cardiac endothelial cells failed the exert protection in mice with myocardial infarction [189]. Therefore, it is very important, to obtain the proper balance of PPAR-α/β/δ activation in the different cardiac cell types to observe beneficial effects on the outcome in ischemic heart disease.

### 3.5. The Modulation of PPARs in Experimental Models of Stroke

PPARs are highly expressed in the brain and play a crucial role in the CNS. It has been shown that PPAR-γ and its coactivator PGC-1α is engaged in cell differentiation and mitochondria biogenesis as well as in neurodegeneration and neuroinflammation [190]. PPAR-α was shown to influence metabolism of amyloid beta precursor protein (APP) and phosphorylation of Tau protein [191]. PPAR-β/δ has a role in differentiation of cells, lipid metabolism and myelination in CNS [192]. Considering that PPARs are involved in protecting the brain against neuroinflammation, neurodegeneration and oxidative stress, the use of PPARs as a target for stroke treatment has been elucidated by many researchers.

Promising results come from clinical trials on patients undergoing stroke who were treated with pioglitazone. In these patients, lowered risk of recurrent stroke and reduced number of cardiovascular deaths were observed [193,194]. In experimental study, mice lacking PPAR-γ and subjected to MCAO exhibited higher neuronal cell death than control mice. Apoptotic cell death was accompanied by an increase in caspase-3 and Bcl-2 associated X protein levels and reinforcement of endoplasmic reticulum (ER) stress [195]. Oleic acid (OA) is endogenous ligand of PPAR-γ released from the brain phospholipids after cerebral ischemia. Song and colleagues [196] showed that OA has a neuroprotective capacity in the mouse model of stroke, which might be related to its anti-inflammatory actions through PPAR-γ. Han and colleagues [197] showed that treatment with the PPAR-γ agonist rosiglitazone, improves long-term white matter integrity after cerebral ischemia, at least, in part, by promoting oligodendrogenesis and facilitating microglial polarization toward the beneficial M2 phenotype. Another study conducted in the rat model of cerebral ischemia has shown that rosiglitazone decreased ischemia-induced levels of TNF-α, IL-1β and IL-6 and it induced ischemia-downregulated IL-10 level [198]. The effects of rosiglitazone were inhibited by PPARγ antagonist GW9662. Moreover, 1, 25-dihydroxyvitamin D3, activating PPAR-γ, can maintain MCAO-disrupted BBB permeability, via PPAR-γ-dependent inhibition of neuro-inflammation [199]. Pan and colleagues [200] showed that a natural anti-oxidant from the Chinese plant *Hopea hainanensis* Malibatol A exerts anti-inflammatory potential in MCAO mice in a PPAR-γ-dependent manner. Another in vivo study indicated that PPARα agonist octadecylpropyl sulfamide reversed the memory and motor deficits, decreased the activation of microglia and astrocytes, and reduced neurodegeneration in mice subjected to hypoxia/ischemia [201].

The only pharmacological treatment against ischemic stroke is rt-PA administration, within 3–4.5 h from the first stroke symptoms. However, delayed rt-PA administration may lead to BBB breakdown and in turn to hemorrhagic transformation. Recently, it has been demonstrated that rosiglitazone prevented MCAO-induced BBB damage and blocked hemorrhagic transformation in delayed tPA-treated mice by activating PPAR-γ [202]. Similarly, pioglitazone prevented hemorrhagic infarction after transient focal ischemia in diabetic mice via inhibition of inflammation and oxidative stress induced by reperfusion [203].

The drug repurposing strategy identifies available drugs exerting cerebral protective effects. Among these, telmisartan, an antihypertensive agent protected rat brain against MCAO via PPAR-γ and PPAR-γ regulated factor *Egr-1* [204]. Raloxifene and bazedoxifene, used in the treatment of postmenopausal osteoporosis, protected neurons against hypoxia partially via up-regulation of PPAR-γ [122]. Mifepristone used for the termination of early pregnancy exerted neuroprotective potential acting as PPAR-γ agonist and inhibiting inflammatory cytokines and metalloproteinases in rats subjected to MCAO [205]. Aleglitazar, a dual PPAR-α/γ agonist, approved for the treatment of diabetes, attenuated MCAO-induced inflammation via inhibition of microglia migration, phagocytosis, and release of cytokines [206]. Another PPAR-α/γ dual agonist, propane-2-sulfonic acid octadec-9-enyl-amide (N15), exhibited neuroprotective potential in mouse model of stroke via stimulation of PPAR-α/γ signaling and inhibiting the activation of the NF-κB, STAT3, and ERK1/2 signaling pathways [207]. Icaraside, an active flavonoid compound used for erectile dysfunction, osteoporosis, dementia and cancer treatment, administered as pre-treatment in rats model of I/R, protected against cerebral injury via up-regulation of PPAR-α and PPAR-γ and inhibition of NF-κB activation [208,209]. The 14 days pre-treatment of rats with fenofibrate and pioglitazone used for dyslipidemia and type 2 diabetes treatment respectively, improved ischemia-induced neurobehavioral dysfunction, reduced cerebral infarct volume, attenuated inflammatory and apoptotic markers and ameliorated histopathological changes in ischemia injured rats [210].

Not only pharmacological modulation of PPARs receptors may lead to neuroprotection in stroke. Li and colleagues [211] demonstrated that transcutaneous auricular vagus nerve stimulation (ta-VNS) exert a strong protective effect in rats after cerebral I/R injury. The authors observed that ta-VNS decreased neuronal injury, reduced infarct volume, and increased angiogenesis via upregulation of PPAR-γ expression in the ischemic cortex. The other group demonstrated an anti-inflammatory action of vagus nerve stimulation in cerebral ischemia/reperfusion rats via PPAR-γ upregulation [212]. Liu and colleagues [213] also showed that electrical stimulation of cerebellar fastigial nucleus protected rat brains against ischemia via PPAR-γ upregulation, attenuation of apoptosis and inflammation.

One of the promising experimental strategies against stroke-induced injury is the use of stem cells, which are able to differentiate into diverse cellular population and to replace dying cells during stroke. It has been demonstrated that in rat model of cerebral ischemia, pioglitazone was able to activate innate stem cells in the subventricular zone (SVZ) and recruitment of bone marrow (GFP^+BM^) stem cells with an increase in PPAR-γ and then increased the expression of *Akt*, *Map2*, and *Vegf* in the cortical peri-infarct area, leading to neurogenesis. Both types of proliferated stem cells migrated from the SVZ into the peri-infarct area and differentiated into mature neurons, glia, and blood vessels [214].

There are also data showing that expression of PPARs is regulated by miRNAs. In rat model of neonatal hypoxic ischemic encephalopathy (HIE), intranasal administration of the PPAR-β/δ agonist GW0742 diminished neuronal death and apoptosis via PPAR-β/δ/miR-17/TXNIP pathway [215]. Moreover, downregulation of *miR-383* upregulated *Pparg* expression and exerted anti-inflammatory and neuroprotective effect in rat model of ischemic stroke [216]. Recently, the role of RXR receptor (PPARs heterodimerization partner) in cerebral diseases was investigated. Mice lacking RXRα in myeloid phagocytes (Mac-RXRα^−/−^) had a worse functional recovery and they developed brain atrophy after tMCAO [217]. Bexarotene, acting via RXR, improved neurological deficits and exerted anti-inflammatory effect partially through PPAR-γ/SIRT6/FoxO3a pathway in a rat model of subarachnoid hemorrhage [218].

## 4. Targeting of Aryl Hydrocarbon Receptor (AhR) as Promising Therapeutic Strategy in Myocardial Infarction and Stroke

Human aryl hydrocarbon receptor (AHR) is located on chromosome 7 (7p15) [219]. For a long time AHR was considered only as a regulator of response to environmental pollutants via induction of P450 cytochromes (CYP1A1, CYP1A2, CYP1B1) involved in detoxification. However, a high degree of conservation among the species and the phenotypic alterations observed in AhR-deficient mice suggest a strong involvement of the AhR in cell physiology [220]. Indeed, the loss of the AhR in mice resulted in malformation of the liver [221], heart [222,223,224] and mammary gland [225], in extensive immune dysfunctions [226,227], modulation of stem cells [228,229], oculomotor deficits and defective optic nerve myelin sheath [230] and neuronal deficits [231,232]. For example, lack of AhR in murine cerebellar neuron precursors led to an impairment of neurogenesis. Whereas, an activation or silencing of the AhR in the heart resulted in inhibition of cardiomyocytes differentiation [233,234,235]. It was also demonstrated that AhR is involved in the regulation of cardiomyocytes apoptosis and inflammation [236,237]. Moreover, expression of AhR increases in necrotic myocardium after myocardial infarction induced by LAD ligation [237].

Several endogenous AhR ligands as 6-formyl (3,2-b) carbazole (FICZ), 2-(10-H-indole-3-carbonyl) thiazole-4-carboxylic acid methyl ester (ITE), tryptophan metabolites including kynurenine and other gut microbial products, and leukotrienes [238,239] have been identified, but their physiological role is still under debate [240].

**Figure 3:**
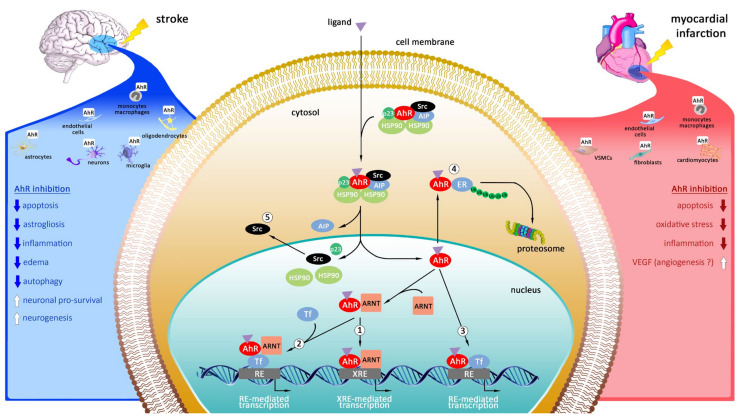
Schematic model representing the cellular localization, the molecular mechanisms and the effects of AhR activation after cerebral and cardiac ischemia. Under unbounded state, AhR is retained in the cytoplasm in an inactive complex. After ligand binding, AIP is released from the complex and the ligand-AhR-HSP90-p23-Scr structure translocates into the nucleus. Inside the nucleus, the ligand-AhR structure is released from the complex and heterodimerizes with the aryl hydrocarbon receptor nuclear translocator (ARNT) and interacts with the xenobiotics response element (XRE) (1), regulating the expression of several phase I and phase II metabolizing enzymes. The ligand-AhR-ARNT (2) and ligand-AhR (3) can interact with other transcription factors (e.g., NF-kB and the estrogen receptor ER), binding to their response elements (RE) and modulating the expression of their target genes. AhR signaling also includes non-genomic pathways: AhR can function as an E3 ubiquitin ligase (4), while the release of the c-Src kinase (5) results in the phosphorylation of multiple targets.

The cellular localization, the mechanisms of action and the protective effects of AhR inhibition during heart and brain ischemia are described on the Figure 3.

### 4.1. Cellular Localization of AhR in the Heart

The study with the use of AhR-KO mice implies that the myocardium could be a target of AhR signaling [241]. AhR-KO mice were characterized by cardiac hypertrophy and cardiomyopathy accompanied by diminished cardiac output [222,242]. Moreover, it has been demonstrated that porcine aorta endothelial cells, vascular smooth muscle cells and cardiac myocytes respond to AhR agonists [243,244,245]. AhR was also found in cardiac fibroblasts [246] and monocytes [247]. In the developing mouse heart (ED13.5 and ED15.5), AhR was detected mainly in the nucleus of endothelial cells lining the internal and external surfaces of the myocardium, endocardium, and epicardium. At the later phase of development (ED18.5), AhR was observed in cytoplasm of cardiac troponin T-positive cardiomyocytes [248].

### 4.2. Cellular Localization of AhR in the Brain

It has been shown that *Ahr* mRNA is present in the mouse brain at the very early developmental stage [249]. *Ahr* was detected in the cerebral cortex especially in innermost cortical layer on ED12.5. On ED18.5, expression was observed in the hippocampus (pyramidal cell layer of the CA1 and CA3, granule cell layer of the DG regions) and in cerebral cortex. Postnatally, the expression of *Ahr* was observed at 3, 7 and 14 days after birth, in the CA1 and CA3 pyramidal cell layers, DG granule cell layer of the hippocampus, in the cerebral cortex, cerebellum (the external granule cell layer on earlier days, the granule cell layer on PND 14), in the granule cell layer of the olfactory bulb and the rostral migratory stream (RMS). In the brain of 12-week-old mice, *Ahr* expression was observed in the hippocampal CA1 and CA3 pyramidal and DG granule cell layers, cerebral cortex, cerebellar and olfactory bulb granule cell layers, and rostral migratory system [249]. *Ahr* was also detected in neurons, astrocytes, microglia, oligodendrocytes [250,251,252,253,254,255,256], monocytes/macrophages [247] and cerebral endothelial cells [250]. Interestingly, higher level of AhR protein in astrocytes was detected in the brain of elderly than young people [257]. This discovery makes AhR even more attractive target for future therapies against stroke, which occurs mainly in older people.

### 4.3. Mechanisms of Action of Aryl Hydrocarbon Receptor

AhR is transcription factor belonging to a superfamily of basic helix-loop-helix/Per-ARNT-Sim (bHLH/PAS). In an inactive state AhR is localized in cytoplasm as part of a complex that consists of a dimer of the 90 kDa heat shock protein (HSP90), AhR-interacting protein (AIP; also known as XAP2), the co-chaperone p23 and the protein kinase SRC. Upon ligand binding, AIP dissociates from the complex and translocates to the nucleus in a transportin-dependent and importin-β-dependent manner [258]. Once in the nucleus, AhR binds to Aryl Hydrocarbon Receptor Nuclear Translocator (ARNT), and the dimer is recruited to xenobiotic response element (XRE) activating transcription of many genes involved not only in response to environment pollutants but also in development of cardiovascular and central nervous system [259,260]. Apart from binding to XRE, AhR can also bind to ERE and regulates gene expression by creating a dimers with estrogen receptors in the absence of their ligands [261]. AhR is able to inhibit ERs activity through the binding to the iXRE in the promoters of ERs target genes, squelching of shared coactivators or increased proteasomal degradation of ERs [18]. AhR controls also NF-κB and signal STAT proteins that play a crucial role in the regulation of the immune responses [224,262].

### 4.4. The Modulation of AhR in Experimental Models of Myocardial Infarction

It is well known that air pollution may increase a risk of development of cardiovascular diseases [263,264]. It has been demonstrated that exposure to particulate matter (PM) pollutants is linked to myocardial infarction, cardiac arrhythmias, increased blood coagulability, atherosclerosis and stroke [265]. Indeed, it has been shown that different manipulation of AhR such as AhR activation, AhR inhibition or AhR knockdown can affect cardiomyocyte differentiation via disruption of AhR-regulated genes e.g., homeobox transcription factors [228,235,266]. Vilahur and colleagues [267] showed a significant increase in AhR expression in pig myocardium after I/R injury, while post-ischemic conditioning inhibited AhR expression thus suggesting the important role of AhR signaling pathway in myocardial injury. Indeed, myocardial infarction induced AhR and AhR-regulated IL-1β and IL-6 in mice heart and a natural flavone baicalin was able to inhibit the myocardial injury and inflammation by decreasing the expression of AhR [237]. Li and colleagues [268] showed dual nature of AhR agonist beta-naphtoflavone (β-NF) in protecting H9C2 cells against OGD. From one side, β-NF reversed OGD/R-induced ROS overproduction, decreased cell death, lactate dehydrogenase release and caspase-3 activity, from another side β-NF, activating AhR, blocked the binding of ARNT to cardioprotective hypoxia-inducible factor (HIF)-1α and in turn inhibited VEGF production and stimulated induction of nitric oxide (NO).

Because of few data on the mechanisms of action of AhR in myocardial infarction, there is an urgent need to unravel the role of AhR signaling pathway during the heart injury.

### 4.5. The Modulation of AhR in Experimental Models of Stroke

There is an increasing body of evidence that blocking of AhR signaling pathway could be a promising strategy in stroke therapy. It has been shown that experimental stroke is followed by an increase of AhR in the murine brain. Pharmacological and genetic inhibition of AhR leads to a strong reduction of infarct volume in mice subjected to MCAO. AhR antagonist TMF increased CREB transcriptional activity, normalizing BDNF levels and decreasing apoptosis by affecting apoptosis-related genes (reduced pro-apoptotic proteins p53 and Puma and increased the anti-apoptotic Bcl-X) after MCAO [269]. Similarly, TMF decreased infarct volume, inhibited astrogliosis, microgliosis and apoptosis in rodent brains undergoing MCAO [231,270]. Furthermore, TMF treatment modulated gene and protein expression related to neurogenesis after stroke, leading an increased proliferation of neural progenitor cells at the ipsilesional neurogenic zones [231]. Tanaka and colleagues [252] showed that AhR antagonist CH223191 inhibited MCAO-increased the expression of *Tnfa* and edema progression, and improved the neurological severity scores in mice. The administration of 3,3’-diidnolylmethane (DIM), a selective AhR modulator, protected hippocampal neurons against hypoxia/ischemia via inhibition of AhR signaling pathway. The neuroprotective action of AhR antagonism against ischemia likely involves an inhibition of apoptosis and autophagy [271,272]. Moreover, an in vivo study confirmed that DIM protected rat pups against perinatal asphyxia via inhibition of AhR and NMDA signaling pathways [273]. The latest data showed that intracerebral hemorrhage in mouse induced the expression of AhR in microglia and neutrophils. DIM attenuated activation of microglia/macrophages and astrocytes and diminished infiltration of neutrophils into the hematoma. DIM also decreased AhR-regulated *Il6* and *Cxcl1* [274]. These results strongly suggest an involvement of AhR in immune cell functions during intracerebral hemorrhage. Although promising experimental evidence on the important role of AhR signaling pathway in stroke pathology was obtained, this topic is still unexplored and requires further research.

## 5. Conclusions

The progress in mechanical therapies (i.e., stenting or mechanical thrombectomy) and the use of thrombolytic drugs in stroke or in myocardial infarction reduced significantly the rate of mortality. However, scientists still have to look for new more efficient and safer drugs that will be able to prolong a short time-window of currently available treatments. Future therapies should focus not only on well-known ischemia-induced mechanisms, but also on new molecular targets (i.e., nuclear receptors) and compounds (i.e., SERMs, SAhRMs), which can help in reduction of infarct-induced damage and significantly improve patient’s life.

## Data Availability

Not applicable.

## Abbreviations

AHR	aryl hydrocarbon receptor
AIP	AHR-interacting protein
Akt (PKB)	protein kinase B
ANP	pro-atrial natriuretic peptide
AP-1	activator protein 1
ARNT	aryl hydrocarbon nuclear translocator
BBB	blood brain barrier
CK-MB	creatine kinase-(muscle–brain)
CNS	central nervous system
CREB	cAMP response element-binding protein
CVDs	cardiovascular diseases
CXCL1	C-X-C Motif Chemokine Ligand 1
CYP1A1	cytochrome P450 1A1
DIM	3,3′-diindolylmethane
DPN	diaryl-propio-nitrile
E2	17β-estradiol
ER	estrogen receptor
ERE	estrogen response element
ERK	extracellular signal-regulated kinase
ERα	estrogen receptor alpha
ERβ	estrogen receptor beta
GPER	G-protein-coupled estrogen receptor
GSK-3β	glycogen synthase kinase 3 beta
HIE	hypoxic ischemic encephalopathy
HSP90	heat shock protein
I/R	ischemia/reperfusion
IL-6	Interleukina 6
JNK	c-Jun N-terminal kinase
KO	knock-out
LAD	left anterior descending
LPS	lipopolysaccharides
LV	left ventricular
MAPK	mitogen-activated protein kinases
MI	myocardial infarction
miRNA	microRNA
MMP	methyl-piperidino-pyrazole
mPTP	mitochondrial permeability transition pore
MSCs	mesenchymal stem cells
MT	mechanical thrombectomy
MTA1	metastasis-associated protein 1
mTOR	mammalian target of rapamycin kinase
NCoR	the nuclear receptor corepressor
NF-κB	nuclear factor-kappa B
NRF-1	nuclear respiratory factor-1
OA	Oleic acid
OVX	ovariectomized
PAI-1	plasminogen activator inhibitor-1
PCG-1	peroxisome proliferator-activated receptor-gamma coactivator 1
PI-3K	3-Phosphatydylinosytol kinase
PKCε	the epsilon isoform of protein kinase C
PLC	phospholipase C
pMCAO	permanent middle cerebral artery occlusion
PPAR	peroxisome proliferator-activated receptor
PPREs	proliferator response elements
PPT	propyl-pyrazole-triol
RACK2	receptor for activated C kinase
ROS	reactive oxygen species
rt-PA	tissue plasminogen activator
RXR	retinoid X receptor
SAHRM	selective aryl hydrocarbon receptor modulator
SERM	selective estrogen receptor modulator
SMRT	silencing mediator of retinoid and thyroid hormone receptor
SOD1	superoxide dismutase
ta-VNS	transcutaneous auricular vagus nerve stimulation
TFAM	mitochondrial transcription factor A
Tg-ERβ	transgenic ERβ
tMCAO	transient middle cerebral artery occlusion
TNF-α	Tumor necrosis factor
VSMCs	vascular smooth muscle cells
XRE	xenobiotic response element
